# Osteoblast Demineralization Induced by Oxidized High-Density Lipoprotein via the Inflammatory Pathway Is Suppressed by Adiponectin

**DOI:** 10.3390/ijms232314616

**Published:** 2022-11-23

**Authors:** Noor Hanisa Harun, Gabriele Ruth Anisah Froemming, Aletza Mohd Ismail, Hapizah Nawawi, Siti Shuhada Mokhtar, Suhaila Abd Muid

**Affiliations:** 1 Department of Biochemistry and Molecular Medicine, Faculty of Medicine, Universiti Teknologi MARA, Sungai Buloh 47000, Selangor, Malaysia; 2 Department of Basic Medical Sciences, Faculty of Medicine and Health Sciences, Universiti Malaysia Sarawak, Kota Samarahan 94300, Sarawak, Malaysia; 3 Department of Pathology, Faculty of Medicine, Universiti Teknologi MARA, Sungai Buloh 47000, Selangor, Malaysia; 4 Institute of Pathology, Laboratory and Forensic Medicine (I-PPerforM), Universiti Teknologi MARA, Sungai Buloh 47000, Selangor, Malaysia

**Keywords:** oxidized high-density lipoprotein (HDL), adiponectin, osteoblast cells, mineralization, inflammatory pathway

## Abstract

Low mineralization activity by human osteoblast cells (HOBs) indicates abnormal bone remodeling that potentially leads to osteoporosis. Oxidation, the most prominent form of high-density lipoprotein (HDL) modification, is suggested to affect bone mineralization through the inflammatory pathway. Adiponectin, which possesses anti-inflammatory activity, is postulated to have the ability to suppress the detrimental effects of oxidized HDL (oxHDL). This study aimed to investigate the effects of HDL before and after oxidation on markers of mineralization and inflammation. The protective effects of adiponectin on demineralization and inflammation induced by oxHDL were also investigated. OxHDL at 100 µg/mL protein had the highest inhibitory effect on mineralization, followed by lower calcium incorporation. OxHDL also had significantly lower expression of a mineralization marker (COL1A2) and higher expression of inflammatory markers (IL-6, TNF-α, and RELA proto-oncogene, NF-κβ (p65)) compared to the unstimulated control group. These findings suggest that oxHDL reduces the mineralization activity of HOBs by increasing the expression of inflammatory markers. Interestingly, co-incubation of adiponectin and oxHDL in HOBs resulted in higher expression of mineralization markers (ALPL, COL1A2, BGLAP, and RUNX2) and significantly reduced all targeted inflammatory markers compared to the oxHDL groups. On the contrary, HDL increased the expression of mineralization markers (COL1A2 and STAT-3) and exhibited lower expression of inflammatory cytokines (IL-6 and TNF-α), proving the protective effect of HDL beyond the reverse cholesterol transport activity.

## 1. Introduction

Bone mineralization is an important process during bone construction and hardening. During the bone mineralization process, calcium and phosphate are accumulated in the mineral vesicle that buds off the osteoblast cells to produce hydroxyapatite crystals. This hydroxyapatite crystal is deposited onto the organic extracellular matrix in an organized manner [1]. Bone mineralization is regulated explicitly by osteoblast cells, one of the bone cell types that lie along the bone surface, which comprises 4–6% of the total resident bone cells [2]. Any disruption during osteoblast differentiation or maturation leads to a lower bone mineralization rate and, in severe cases, may lead to osteoporosis. Osteoporosis is a “silent disease”, which causes an individual to be unaware of their bones gradually becoming weaker and brittle until they eventually break. Osteoporosis develops when there is a decrement in bone mineral density (BMD), in which osteoporotic patients usually have a T-score of −2.5 or less when measured by a DEXA (dual-energy X-ray absorptiometry) scan [3]. About 18.3% of the world population suffers from osteoporosis, which affects the patient’s quality of life and increases the burden on government agencies and medical institutes in various fields [4,5]. Much effort in terms of caregiver time and medical costs is required for acute rehabilitative care following osteoporotic fractures [6]. Therefore, intensive research on preventing the occurrence of osteoporosis at the very first and most basic level, which is the cell osteoblast, is crucial.

A histomorphometry study by Byers et al. in 1997 found that most osteoporotic patients had a significantly lower number of osteoblast cells, and these osteoblasts have a lower mineralization rate, indicating a lower percentage of active osteoblasts in patients and leading to reduced efficiency of the active osteoblasts [7]. This shows that osteoblast dysfunction, characterized by a lower mineralization rate, is involved in the pathogenesis of osteoporosis. Dyslipidemia, specifically a low level of high-density lipoprotein (HDL), is one of the strongest candidates that could disturb bone mineralization activity as osteoporosis has been found to co-exist in rodents with hyperlipidemia [8].

Pro-inflammatory processes are suggested to play an important role in inhibiting osteogenic differentiation of mesenchymal stem cells (MSCs) and osteoblast differentiation, leading to disruption of bone-forming activities [9]. Osteoblasts’ bone formation activity is crucial in replacing new bone tissue at the site where osteoclasts have removed the old and damaged bone tissue to ensure no net changes in the bone mass density. The disruption in the bone-forming activity interrupts the balanced tight coupling process between osteoblasts and osteoclasts in the bone remodeling cycle, leading to bone loss [10]. Consequently, the bone loses its strength and becomes prone to frequent fractures (breaks), which is one of the important signs of osteoporosis.

Previous studies reported the presence of oxidized lipids and lipoproteins that induce inflammation in osteoblasts, causing these cells to malfunction [11]. The accumulation of oxidized lipids or lipoproteins also induces oxidative stress in the bone, commonly observed in osteoporotic and aging human bone. Interestingly, oxidized lipids are found in the bone marrow of hyperlipidemic mice. The in vivo study reported that mice fed an atherogenic high-fat diet had reduced bone mineralization activities with higher osteoclastogenesis activity. The bone marrow cells showed suppressed expression of osteocalcitonin, which potentially lead to the inhibition of osteoblastic differentiation [12]. The induction of minimally oxidized low-density lipoprotein (oxLDL) and oxidized 1-palmitoyl-2-arachidonoyl-sn-glycero-3-phosphorylcholine (oxPAPC), including 8-iso prostaglandin E2 isoprostanes, caused inhibition of alkaline phosphatase (ALP) activity and calcium uptake in MC3T3-E1 pre-osteoblast cells [11]. Moreover, oxLDL suppressed the secretion of Cbfa1/RUNX2 transcription and osteopontin in inorganic-phosphate-induced UMR106 rat osteoblasts, which reduced the mineralization ability of these cells [13].

HDL (high-density lipoprotein) is another lipoprotein that is also prone to oxidation, especially when exposed to oxidative stress, inflammation, hyperglycemia-induced advanced glycation end-products, and the presence of reactive oxygen species. HDL is known to have many beneficial activities in cardioprotection. However, oxidation causes the components of HDL to change in terms of the quantity and quality, and this HDL later becomes dysfunctional [14,15]. This dysfunctional HDL loses its anti-inflammatory and antioxidative properties, thus impairing its anti-atherogenic activities and other vital functions. Furthermore, dysfunctional HDL starts to behave like pro-inflammatory and pro-atherogenic molecules [16]. However, studies on the role of HDL in bone metabolism are scarce. There is a possibility that the presence of oxidized HDL (oxHDL) could interrupt bone mineralization. A study performed on mice with ApoA-I-deficiency showed a reduction in BMD, accompanied by lower secretion of osteoblast-related markers such as RUNX2, Osterix, and Col1α [17]. ApoA-I is the major protein component of HDL [18]. Moreover, another study by Martineau’s research group also showed the elevated plasma level of HDL-cholesterol in Scarb1-deficient mice, higher trabecular bone volume, and a greater osteoblast number and function compared to the wild-type mice [19]. Scarb1 is a gene that encodes Scavenger receptor class B, type I (SRBI), an HDL receptor that regulates reverse cholesterol transport (RCT). Deficiency of the Scarb1 gene is commonly associated with the elevation of adrenocorticotropic hormone (ACTH) levels, which have anabolic effects on osteoblast cells [20,21]. Perhaps, HDL might play an important role in preserving the biological activity of osteoblast cells, thus maintaining normal bone homeostasis. However, its actual mechanisms are still unclear and require further research.

Osteoporosis is also associated with obesity. The secretion of myokines, osteokines, and adipokines is the main bridge of the crosstalk between bone tissue and fat tissue [22]. For instance, adipokines released by the adipose tissues, such as leptin, resistin, adiponectin, and TNFα, could modulate bone metabolism [22]. Interestingly, one of the common features observed in osteoporosis and obesity is the lack or lower secretion of adiponectin. Adiponectin is one of the major adipokines predominantly secreted by adipocytes in the white adipose tissues [23]. Interestingly, the secretion of adiponectin is inversely correlated with body mass and total body fat. Adiponectin has been postulated to have beneficial activity in preventing osteoporosis by enhancing osteogenesis, leading to increased bone formation activity and, at the same time, inhibiting bone resorption activity [24]. In osteoporosis, the osteogenic cell population in the bone marrow stroma is declining while adipocyte cell numbers are increasing [25]. The incubation of murine mesenchymal progenitor cells with adiponectin showed a positive elevation of osteoblastic differentiation. This was accompanied by increased expression of some osteogenic marker genes [26]. Moreover, induction of recombinant adiponectin into normal human osteoblasts elevates alkaline phosphatase (ALP) activity, osteocalcin, and collagen type I production, accompanied by the intense formation of mineralized nodules [27]. This evidence shows that adiponectin plays an important role in protecting the regulation of bone formation activity. However, it is still unclear whether adiponectin could protect osteoblast cells from the detrimental effects of oxHDL.

Therefore, the aims of this study were to investigate the effects of HDL before and after oxidation on bone mineralization to examine whether oxHDL can induce inflammation in primary human osteoblasts and elucidate its underlying mechanism and, finally, investigate the ability of adiponectin in suppressing the detrimental effects of oxHDL.

## 2. Results

### 2.1. OxHDL Reduces the Formation of Mineral Nodules and Calcium Incorporation in HOBs

**Figure 1:**
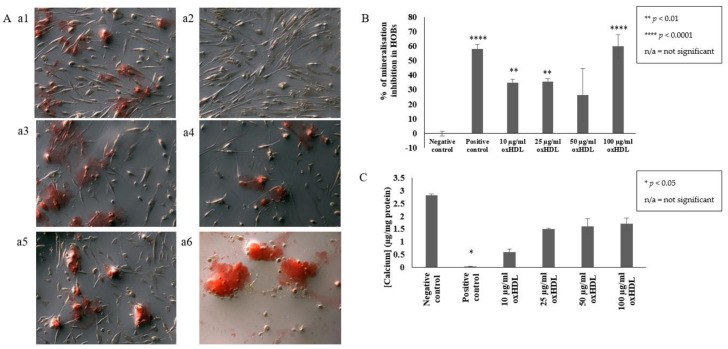
Effects of oxHDL on mineralization and calcification in HOBs. (**A**) Cell morphology and formation of mineral nodules. The pictures were captured at 10× magnification using a phase-contrast microscope (Olympus IX81 microscope). HOBs were incubated with (**a1**) culture medium (negative control); (**a2**) inorganic pyrophosphate (positive control); and (**a3**) 10, (**a4**) 25, (**a5**) 50, and (**a6**) 100 μg/mL protein oxHDL, for 14 days. The medium was changed for every 3 days. HOBs were stained with 2% alizarin red for the detection of mineral nodules indicated by the presence of red color. (**B**) The percentage of mineralization inhibition in HOBs was quantified by measuring the concentration of Alizarin red staining in the cells. (**C**) Calcium deposition was analyzed using a calcium colorimetric assay. Data are expressed as mean ± SEM of duplicate readings. * *p* < 0.05, ** *p* < 0.01, **** *p* < 0.0001 compared to the negative control. Non-available (n/a) indicates not significant.

In this study, alizarin red was used to stain the mineral nodules produced by the HOBs. The red stain observed during microscopic examination (Figure 1A) indicates the region formation of mineral nodules. This red stain was extracted for quantitative analysis. Quantitative analysis confirmed the presence of 10, 25, and 100 µg/mL protein oxHDL significantly inhibited the formation of mineral modules in HOBs compared to cells grown in culture media only (35 ± 2.2% (*p* < 0.01), 35.7 ± 2.1% (*p* < 0.01), 59.7 ± 8.2% (*p* < 0.0001), respectively, vs. 0.0 ± 1.6%) (Figure 1B). Furthermore, cells incubated with 100 µg/mL protein oxHDL showed the highest inhibition, even higher than the positive control, inorganic pyrophosphate (59.7 ± 8.2% (*p* < 0.0001) vs. 58.2 ± 3.1% (*p* < 0.0001)). Only 50 µg/mL protein oxHDL did not significantly inhibit the formation of mineral nodules (26.5 ± 18%), but it did show a decremental trend. A trend of a reduction in calcium deposition was also observed in the HOBs cells treated with 10, 25, 50, and 100 µg/mL protein oxHDL (0.6 ± 0.11 µg/mg protein, 1.5 ± 0.02, 1.6 ± 0.31, and 1.7 ± 0.24 µg/mg protein, respectively) (Figure 1C). Microscopic examination of cells incubated with 10, 25, and 100 µg/mL protein oxHDL also showed a reduction in cell counts. The cell morphology of HOBs incubated with 100 µg/mL protein oxHDL also changed, and some of the cells looked like they were bursting. The concentration of 100 μg/mL oxHDL was selected for an investigation of the gene expression of inflammation and osteogenic markers in HOBs.

### 2.2. Effect of HDL, oxHDL, Adiponectin, and Co-Incubation of oxHDL with Adiponectin on the Expression of Bone Mineralization Markers (RUNX2, ALPL, COL1A2, BGLAP) 

**Figure 2:**
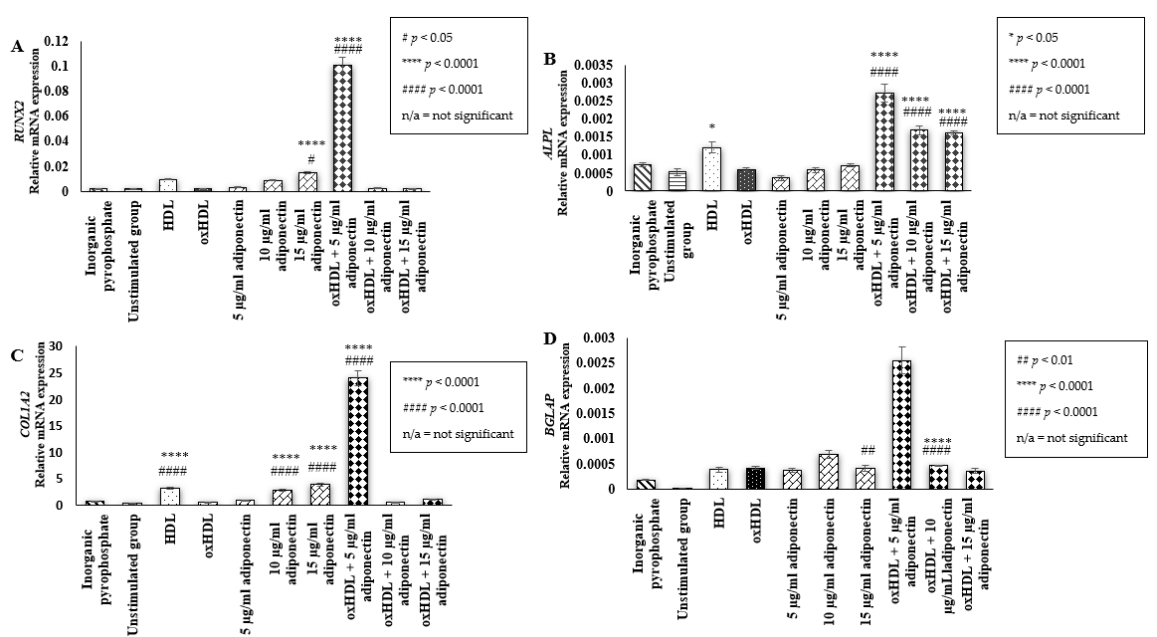
Effects on bone-associated markers in HOBs. Effects of (i) inorganic pyrophosphate (positive control), (ii) culture medium (negative controls, denoted as the unstimulated group), (iii) HDL (100 μg/mL protein), (iv) oxHDL (100 μg/mL protein), (v) adiponectin (5, 10, and 15 μg/mL), and (vi) the combination of oxHDL (100 μg/mL) with adiponectin (5, 10, and 15 μg/mL) on mRNA gene expression of (**A**). RUNX2, (**B**). ALPL, (**C**). COL1A2, and (**D**). BGLAP in HOBs. Data are expressed as the mean ± SEM of 3 experiments. * *p* < 0.05, **** *p* < 0.0001, ^#^ *p* < 0.05, ^##^ *p* < 0.01, ^####^ *p* < 0.0001. Non-available (n/a) indicates not significant. * Indicates the comparison with the oxHDL group; # Indicates the comparison with the unstimulated group. GAPDH and HPRT1 genes were used as the reference gene.

RUNX2 plays an important role during osteoblast differentiation from mesenchymal stem cells to pre-osteoblast and intermediate osteoblast cells [28]. In this study, HOBs cells incubated with 15 µg/mL adiponectin alone (0.015 ± 0.001 relative mRNA expression (*p* < 0.05, *p* < 0.0001)) and co-incubation of oxHDL with 5 µg/mL adiponectin (0.1 ± 0.006 relative mRNA expression (*p* < 0.0001)) showed an increment in RUNX2 gene expression when compared to the unstimulated (0.002 ± 0.000 relative mRNA expression) and oxHDL group (0.002 ± 6.7 × 10^−5^ relative mRNA expression) (Figure 2A). In general, two main predictions can be made: firstly, RUNX2 might not be affected by the presence of HDL and oxHDL; secondly, these cells do not produce RUNX2 as the cells used in this experiment were mature adult human osteoblast cells. Therefore, several bone matrix proteins were selected as target markers to understand the effect of all treatments on the mineralization activity of HOBs. Bone matrix proteins are important in anchoring calcium to the matrix during the bone mineralization process. 

One of the markers is ALPL, a gene coding for alkaline phosphatase protein. In this study, the cells treated with oxHDL (0.0006 ± 5.1 × 10^−5^ relative mRNA expression) and adiponectin alone, 5–15 µg/mL (5 µg/mL adiponectin: 0.0004 ± 7.2 × 10^−5^ relative mRNA expression, 10 µg/mL adiponectin: 0.0006 ± 7.3 × 10^−5^ relative mRNA expression, and 15 µg/mL adiponectin: 0.0007 ± 5.3 × 10^−5^ relative mRNA expression), did not have any effect on the mRNA expression of ALPL when compared to the unstimulated group (0.0005 ± 0.0001 relative mRNA expression) (Figure 2B). Unexpectedly, cells treated with the co-incubation of oxHDL and adiponectin (5–15 µg/mL) showed significant elevation in ALPL mRNA expression (oxHDL + 5 µg/mL adiponectin: 0.0027 ± 0.0002 relative mRNA expression (*p* < 0.0001), oxHDL + 10 µg/mL adiponectin: 0.0017 ± 0.0001 relative mRNA expression (*p* < 0.0001), and oxHDL + 15 µg/mL adiponectin: 0.0016 ± 5.5 × 10^−5^ relative mRNA expression (*p* < 0.0001)) compared to oxHDL alone. Cells treated with HDL had a neutral effect on HOBs in terms of ALPL mRNA expression compared to the unstimulated group (0.0012 ± 0.0002 relative mRNA expression vs. 0.0005 ± 0.0001 relative mRNA expression), but it was significantly higher than cells treated with oxHDL.

Moreover, this study also measured the mRNA expression of COL1A2, which codes for the synthesis of the α2 chain, one of the collagen chains intertwined with the other two α1 chains to form collagen 1 (Figure 2C). In this study, oxHDL did not affect the mRNA expression of COL1A2. Incubation of cells with oxHDL and the lowest concentration of adiponectin (5 µg/mL) showed the highest mRNA secretion of COL1A2 compared to oxHDL alone (24 ± 1.4 relative mRNA expression vs. 0.499 ± 0.025 relative mRNA expression (*p* < 0.0001)). In addition, cells stimulated with 10 and 15 µg/mL adiponectin also showed significantly higher mRNA secretion of COL1A2 when compared to the unstimulated group (10 µg/mL adiponectin: 2.8 ± 0.1 relative mRNA expression (*p* < 0.0001), 15 µg/mL adiponectin: 3.9 ± 0.3 relative mRNA expression, respectively (*p* < 0.0001), and unstimulated: 0.28 ± 0.007 relative mRNA expression). Incubation of cells with HDL also showed a significantly higher mRNA expression of COL1A2 compared to the unstimulated group and the oxHDL group (HDL: 3.2 ± 0.1 relative mRNA expression (*p* < 0.0001) vs. unstimulated: 0.28 ± 0.007 relative mRNA expression and oxHDL: 0.499 ± 0.025 relative mRNA expression).

In addition, cells treated with 10 µg/mL adiponectin alone (0.0007 ± 7.74 × 10^−5^ relative mRNA expression (*p* < 0.01)) showed higher mRNA expression of bone gamma-carboxyglutamate protein (BGLAP), which encodes for osteocalcin, compared to the unstimulated group. Moreover, cells co-incubated with oxHDL and 5 µg/mL adiponectin (0.0025 ± 0.0003 relative mRNA expression (*p* < 0.0001)) showed significantly higher mRNA secretion of BGLAP compared to the oxHDL group and unstimulated group (Figure 2D). Treatment of cells with HDL, oxHDL, and other concentrations of adiponectin (either incubated alone or with oxHDL) did not show any effect on the mRNA secretion of BGLAP.

### 2.3. Effect of HDL, oxHDL, Adiponectin, and Co-Incubation of oxHDL with Adiponectin on the Expression of Osteoblastic Transcription Factor (STAT-3 and PPAR-α)

**Figure 3:**
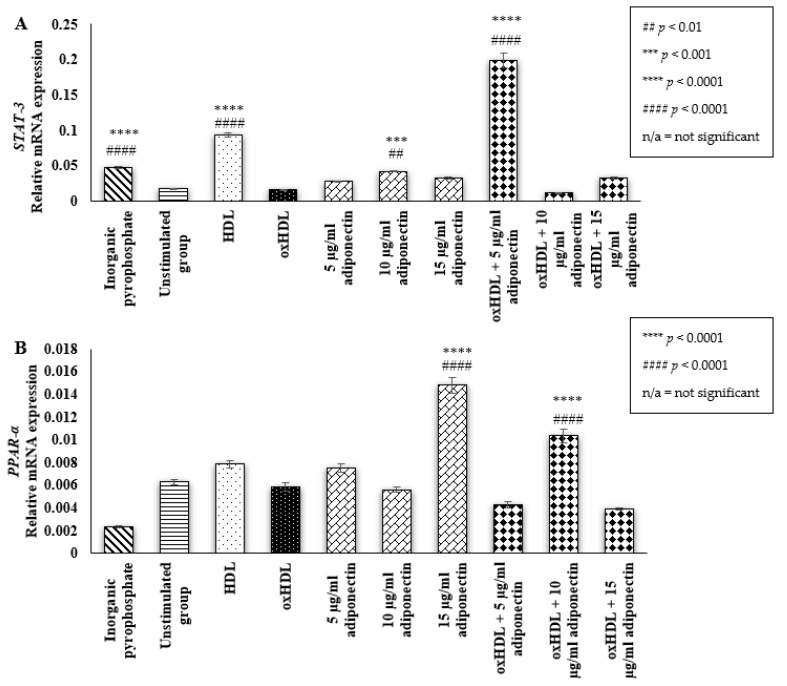
Effects of (i) Inorganic pyrophosphate (positive control), (ii) culture medium (negative control, denoted as the unstimulated group), (iii) HDL (100 μg/mL protein), (iv) oxHDL (100 μg/mL protein), (v) adiponectin (5, 10, and 15 μg/mL), and (vi) the combination of oxHDL (100 μg/mL) with adiponectin (5, 10, and 15 μg/mL) on mRNA gene expression of (**A**). STAT-3 and (**B**). PPAR-α in HOBs. Data are expressed as the mean ± SEM of 3 experiments. *** *p* < 0.001, **** *p* < 0.0001, ^##^ *p* < 0.01, ^####^ *p* < 0.0001. Non-available (n/a) indicates not significant. * Indicates the comparison with the oxHDL group; # Indicates the comparison with the unstimulated group. GAPDH and HPRT1 genes were used as the reference genes.

STAT-3 is one of the important transcription factors involved in stimulating the bone formation activity of osteoblast cells [29]. Interestingly, the presence of HDL (0.093 ± 0.003 relative mRNA expression (*p* < 0.0001)), and 10 µg/mL of adiponectin (0.042 ± 0.001 relative mRNA expression (*p* < 0.01)) resulted in a significantly higher mRNA expression of STAT-3 compared to the unstimulated group (0.017 ± 0.0006 relative mRNA expression) (Figure 3A). The co-incubation of a low concentration of adiponectin (5 µg/mL) with oxHDL also exhibited higher STAT-3 mRNA expression compared to the unstimulated and oxHDL groups (5 µg/mL adiponectin: 0.2 ± 0.01 vs. unstimulated: 0.017 ± 0.0006 and oxHDL: 0.05 ± 0.001, respectively (*p* < 0.0001)). These results showed that HDL and adiponectin might have the ability to increase the secretion of STAT-3 in HOBs. However, oxHDL did not have any effects on the expression of STAT-3 in HOBs.

The expression of PPAR-α was also measured as a previous study demonstrated the involvement of PPAR-α in pre-osteoblast cell maturation [30]. Similar to STAT-3, oxHDL did not have any effects on the mRNA expression of PPAR-α when compared to the unstimulated group (0.0059 ± 0.0004 relative mRNA expression vs. 0.0063 ± 0.0002 relative mRNA expression (*p* < 0.0001)) (Figure 3B). The presence of 10 µg/mL adiponectin during co-incubation with oxHDL increased mRNA expression of PPAR-α when compared to the oxHDL group (0.01 ± 0.0006 relative mRNA expression vs. 0.0059 ± 0.0004 relative mRNA expression (*p* < 0.0001)). In addition, cells incubated with the highest concentration of adiponectin alone (15 µg/mL) also showed significantly higher mRNA expression of PPAR-α compared to the unstimulated group (0.015 ± 0.0007 relative mRNA expression vs. 0.0063 ± 0.0002 relative mRNA expression (*p* < 0.0001)). Cells treated with HDL only showed a trend of increment in the mRNA expression of PPAR-α when compared to the unstimulated group (0.0079 ± 0.0003 relative mRNA expression vs. 0.0063 ± 0.0002 relative mRNA expression (*p* < 0.0001)).

### 2.4. Effects of HDL, oxHDL, Adiponectin, and Co-Incubation of oxHDL with Adiponectin on the Expression of Inflammatory Markers (IL-6, TNF-α, RELA Proto-Oncogene NF-κβ Subunit (p65), and CREB1)

**Figure 4:**
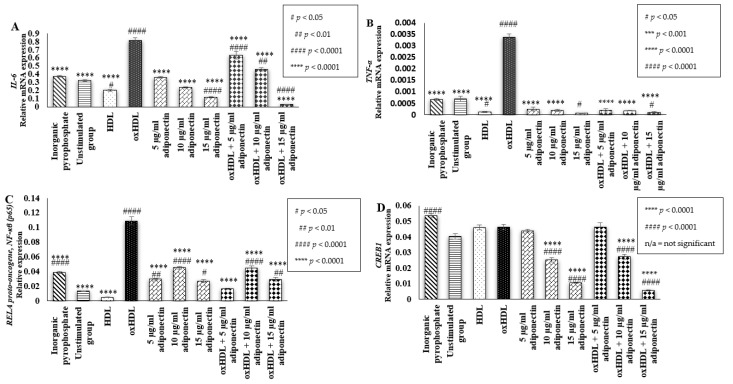
Effects of (i) inorganic pyrophosphate (positive control), (ii) culture medium (negative control, denoted as the unstimulated group), (iii) HDL (100 μg/mL protein), (iv) oxHDL (100 μg/mL protein), (v) adiponectin (5, 10, and 15 μg/mL), and (vi) the combination of oxHDL (100 μg/mL) with adiponectin (5, 10, and 15 μg/mL) on mRNA gene expression of (**A**). IL-6, (**B**). TNF-α, (**C**). RELA proto-oncogene, NF-κβ (p65), and (**D**). CREB1 in HOBs. Data are expressed as the mean ± SEM of 3 experiments. *** *p* < 0.001, **** *p* < 0.0001, ^#^ *p* < 0.05, ^##^ *p* < 0.01, ^####^ *p* < 0.0001. Non-available (n/a) indicates not significant. * Indicates the comparison with the oxHDL group; # Indicates the comparison with the unstimulated group. GAPDH and HPRT1 genes were used as the reference genes.

Inhibition of osteoblast differentiation and maturation by pro-inflammatory cytokines is one of the crucial factors that disturbs a balanced bone remodeling process, leading to bone loss [31]. In this study, it was found that the incubation of oxHDL in HOBs elevated the gene expression of the pro-inflammatory cytokine IL-6 when compared to the unstimulated groups (0.81 ± 0.03 relative mRNA expression vs. 0.32 ± 0.008 relative mRNA expression (*p* < 0.0001)) (Figure 4A). The presence of adiponectin (5–15 µg/mL) during co-incubation with oxHDL showed significant lower mRNA expression of IL-6 compared to the cells treated with oxHDL only (oxHDL + 5 μg/mL adiponectin: 0.63 ± 0.05 relative mRNA expression, oxHDL + 10 μg/mL adiponectin: 0.46 ± 0.02 relative mRNA expression and oxHDL + 15 μg/mL adiponectin: 0.03 ± 0.002 relative mRNA expression, respectively, vs. oxHDL: 0.81 ± 0.03 relative mRNA expression (*p* < 0.0001)). This suggests that adiponectin has a protective effect against the upregulation of IL-6 heightened by oxHDL. Furthermore, a high concentration of adiponectin (15 µg/mL) showed significantly lower IL-6 mRNA expression compared to the unstimulated group (0.12 ± 0.008 relative mRNA expression vs. 0.32 ± 0.008 relative mRNA expression (*p* < 0.0001)). Incubation of cells with HDL alone showed a significantly lower mRNA expression of IL-6 (0.2 ± 0.01 relative mRNA expression) compared to the unstimulated group (*p* < 0.05) and oxHDL group (*p* < 0.0001).

TNF-α is another inflammatory cytokine measured in this study. The presence of oxHDL significantly induced higher mRNA expression of TNF-α compared to the unstimulated and HDL groups (oxHDL: 0.003 ± 0.0001 relative mRNA expression (*p* < 0.0001) vs. unstimulated: 0.0007 ± 0.0001 and HDL: 0.00012 ± 2.27 × 10^−5^) (Figure 4B). This effect of oxHDL was suppressed by the presence of adiponectin (5–15 µg/mL) during co-incubation as the cells exhibited significantly lower mRNA expression of TNF-α compared to the cells treated with oxHDL only (oxHDL + 5 μg/mL adiponectin: 0.00019 ± 7.4 × 10^−5^ relative mRNA expression (*p* < 0.0001), oxHDL + 10 μg/mL adiponectin: 0.00018 ± 1.9 × 10^−5^ relative mRNA expression (*p* < 0.0001) and oxHDL + 15 μg/mL adiponectin: 0.0001 ± 3.4 × 10^−5^ relative mRNA expression (*p* < 0.0001), respectively, vs. oxHDL: 0.0034 ± 0.0002 relative mRNA expression). The highest concentration of adiponectin (15 µg/mL) also showed significantly lower TNF-α mRNA expression compared to the unstimulated group (0.0001 ± 3.4^−5^ vs. 0.0007 ± 0.0001 (*p* < 0.05)). Cells treated with HDL also showed significantly lower TNF-α mRNA expression (0.00012 ± 2.27 × 10^−5^) compared to the unstimulated group, suggesting adiponectin and HDL could have a protective effect in terms of TNF-α mRNA expression. 

The finding from this study suggests that the increment in inflammatory cytokines by oxHDL is via RELA proto-oncogene NF-ĸβ subunit (P65) activation (Figure 4C). the RELA proto-oncogene NF-ĸβ subunit (p65) is the gene that codes for NF-ĸβ (p65) protein. It shows that oxHDL showed a higher elevation of RELA proto-oncogene NF-ĸβ subunit mRNA expression when compared to the unstimulated and HDL groups (oxHDL: 0.11 ± 0.005 relative mRNA expression (*p* < 0.0001) vs. unstimulated: 0.01 ± 0.0004 relative mRNA expression and HDL: 0.005 ± 0.0001 relative mRNA expression). This effect of oxHDL might be suppressed by the presence of adiponectin because significantly lower mRNA expression of the RELA proto-oncogene NF-ĸβ subunit was observed during co-incubation of 5 to 15 µg/mL adiponectin with oxHDL compared to the cells treated with oxHDL alone (oxHDL + 5 µg/mL adiponectin: 0.02 ± 0.0007 relative mRNA expression (*p* < 0.0001), oxHDL + 10 µg/mL adiponectin: 0.04 ± 0.003 relative mRNA expression (*p* < 0.0001) and oxHDL + 15 µg/mL adiponectin: 0.03 ± 0.0025 relative mRNA expression, respectively (*p* < 0.0001), vs. oxHDL: 0.11 ± 0.0054 relative mRNA expression). However, cells incubated with adiponectin alone (5–15 µg/mL) showed higher RELA proto-oncogene NF-ĸβ subunit mRNA expression compared to the unstimulated group (5 µg/mL adiponectin: 0.03 ± 0.0008 relative mRNA expression (*p* < 0.01), 10 µg/mL adiponectin: 0.05 ± 0.002 relative mRNA expression (*p* < 0.0001), and 15 µg/mL adiponectin: 0.03 ± 0.002 relative mRNA expression (*p* < 0.05), respectively, vs. unstimulated: 0.01 ± 0.0004 relative mRNA expression), and the values were significantly lower than cells treated with oxHDL (0.11 ± 0.0055 relative mRNA expression). 

This study also measured the expression of the cAMP-responsive element-binding protein 1 (CREB) gene coding for the CREB proteins, a nuclear factor involved in the cyclic AMP signaling pathway. Activation of the cAMP signaling pathway usually correlates with a decline in mineralization and osteogenesis activity in differentiated osteoblasts [32]. However, in this study, the incubation of oxHDL and HDL in the HOBs also did not affect the mRNA expression of CREB1 compared to the unstimulated group. Induction of adiponectin (5–15 µg/mL), either when incubated alone (5 µg/mL adiponectin: 0.044 ± 0.0009 relative mRNA expression, 10 µg/mL adiponectin: 0.025 ± 0.0015 relative mRNA expression and 15 µg/mL adiponectin: 0.0105 ± 0.0005 relative mRNA expression, respectively) or co-incubated with oxHDL (oxHDL + 5 µg/mL adiponectin: 0.046 ± 0.0026 relative mRNA expression, oxHDL + 10 µg/mL adiponectin: 0.027 ± 0.0015 relative mRNA expression, and oxHDL + 15 µg/mL adiponectin: 0.0058 ± 0.0004 relative mRNA expression), showed a dose-dependent decrement in CREB1 mRNA expression.

## 3. Discussion

A reduction in mineralization activity in HOBs indicates disruption of bone formation activity. It is one of the crucial factors contributing to the unbalanced bone remodeling process as when lost bone fails to be replaced, it may lead to osteoporosis [33]. Instead of producing a complete bone structure, HOBs remove the mineral nodules or matrix vesicles, which serve as the site for accumulating phosphate and calcium to produce hydroxyapatite crystal [1]. Later, this crystallized hydroxyapatite is released, propagated into the extracellular matrix, and deposited between the collagen fibrils on the top of the osteoid, which is required for the bone hardening process [34]. Therefore, the decrement in matrix vesicles stained by alizarin red observed during exogenous induction of oxHDL in this study indicated that the HOBs lost some of their physiological function. Furthermore, Ca^2+^ incorporation into matrix vesicles by the annexin calcium channel and other calcium-binding phospholipids is a crucial step in the formation of hydroxyapatite crystals. A reduction in calcium incorporation was also observed in UMR106 rat osteoblasts during exogenous induction with Cu^2+^-induced oxLDL, which later disturbed the osteoblast protein markers, RUNX2 and OPN [13]. 

In this study, the reduction in the mineralization activity in HOBs by oxHDL might be due to the decrease in COL1A2 expression. COL1A2 is the gene that codes for collagen type 1 protein. This protein is the most dominant constituent of the collagenous organic matrix in bone, which provides the site for hydroxyapatite crystal deposition. Therefore, with the presence of oxHDL, it might reduce the collagen cross-linking and repress the deposition of the crystallized matrix. Subsequently, it might lead to bone mineralization activity impairment, which is commonly observed in patients diagnosed with senile osteoporosis and osteogenesis imperfecta [17]. 

The findings form this study suggest that the inhibitory activity of oxHDL towards the mineralization of HOBs might be via the inflammatory pathway. Inflammation is a well-known culprit that disturbs the balanced bone remodeling process by inducing excessive osteoclast differentiation and inhibiting osteoblast maturation, leading to osteoporosis [35,36]. Patients with chronic inflammatory diseases such as rheumatoid arthritis usually have abnormal bone loss and bone formation activities in the inflamed joints, leading to osteoporosis in the axial and appendicular skeleton [31]. A previous study showed that the exogenous induction of TNF-α suppressed the RUNX2 expression in pluripotent progenitor cells and prevented these cells from differentiating into osteoblast cells [1] while the binding of IL-6 to its receptor, rsIL-6R, inhibits mineralization of MC3T3-E1 cells and primary murine calvaria osteoblasts by reducing the expression of ALP, RUNX2, Osterix, and OCN [37]. In this study, a reduction in HOB mineralization activity by oxHDL was concomitant with increased elevation of the pro-inflammatory cytokines, IL-6 and TNF-α. Pro-inflammatory cytokines have been proven to disturb the balance between osteoclast and osteoblast activity, typically resulting in a net loss of bone. Similarly, in a previous study, the presence of oxLDL in an atherosclerotic murine model stimulates a pro-inflammatory response towards the bone, which leads to a loss of bone mass [38]. In this study, the increment in inflammatory cytokines by oxHDL was followed by an increase in the mRNA expression of RELA proto-oncogene and NF-ĸβ subunit (p65). The activation of NF-ĸβ plays an essential role in inflammation and is usually involved in metabolic syndrome [39]. Specific inhibition of NF-ĸβ in differentiated mice osteoblasts significantly maintains bone formation activity, thus preventing bone loss [35]. In addition, the osteoblastic differentiation of mesenchymal C2C12 cells (an immortalized mouse myoblast cell line) was enhanced when the synthesis of NF-ĸβ (p65) was inhibited [40]. NF-ĸβ acts in an autocrine manner, in which the activation of the NF-ĸβ signaling pathway stimulated by pro-inflammatory cytokines secreted during the inflammation state in turn promotes the secretion of cytokines. Activated NF-ĸβ intensifies the inflammatory reaction, which is beyond the initial stimulus, as found in rheumatoid arthritis patients [41]. Therefore, this current study postulated that the high expression of IL-6 and TNF-α induced by oxHDL promotes the activation of the NF-ĸβ signaling pathway. A previous study also found that the TNF-induced inhibition of the TGFβ-mediated Smad signaling pathway was attenuated by the suppression of NF-ĸβ. TGFβ-mediated Smad signaling is an important pathway involved in stimulating differentiation and mineralization of pre-osteoblasts [42]. It showed that the activation of the NF-ĸβ signaling pathway mediated by pro-inflammatory cytokines interrupts normal physiological osteoblast activity. Based on all the results obtained, this study postulates that the elevation of inflammatory cytokines (IL-6 and TNF-α) by oxHDL causes NF-ĸβ activation. Consequently, some of the important bone matrix proteins (ALPL and COL1A2) required for the formation of hydroxyapatite crystallized matrix vesicles and deposition to form hard bone are suppressed, causing a significant reduction in HOB mineralization.

Interestingly, this study found that adiponectin showed protective effects by suppressing the activation of NF-ĸβ during its co-incubation with oxHDL, which then reduced the secretion of pro-inflammatory cytokines (IL-6 and TNF-α). The suppression of NF-ĸβ inflammatory pathways may be due to the high mRNA expression of PPAR-α induced by adiponectin when incubated alone and during co-incubation with oxHDL. PPAR-α has been known to have inhibitory effects on NF-ĸβ activity, resulting in decreased secretion of IL-6 [43]. In addition, the induction of adiponectin in HOBs reduced the secretion of the CREB1 gene. There are conflicting facts regarding the actual role of CREB and cAMP in osteoblast cells. A study carried out by Zhang et al. (2017) showed that the elevation of the intracellular cAMP level activates phosphorylation of the cAMP response element-binding protein (CREB), which then promotes the secretion of Osterix in MC3T3-E1 (mouse osteoblast) [44]. On the other hand, a study carried out by Nishihara et al. (2018) indicated that cAMP reduced the mineralization activity of osteoblast-like cell lines (TMS-12 cells and MC3T3E1 cells) and promoted phenotypic changes in these cells [45]. In this study, the reduction in CREB1 secretion was predicted to retain the mineralization activity of the HOBs and protect the phenotype of the cells from changing. The mRNA expression of STAT-3 was also elevated when the cells were treated with adiponectin alone and during co-incubation with oxHDL. STAT-3 has been confirmed to play an important role in osteoblast differentiation and bone formation [46]. A study carried out by Itoh et al. (2006) proved that mice with osteoblast-specific disruption of the STAT-3 gene had a lower bone formation rate and showed an osteoporotic phenotype. Perhaps, adiponectin promotes osteoblast differentiation through the activation of the STAT-3 signaling pathway, which requires further studies. This might explain why all the mineralization markers were increased when the cells were incubated with adiponectin (5 µg/mL) during co-incubation with oxHDL. Furthermore, a study carried out in primary osteoblast cells isolated from human trabecular bone found that the induction of recombinant adiponectin in these cells elevated the activity of ALP and enhanced the secretion of OCN and collagen type 1, which then increased mineralization [27]. 

This study also found that before undergoing oxidation, HDL could have some beneficial effects on HOBs. The mRNA expression of COL1A2 and STAT-3 is increasing. There is no solid explanation regarding the protective activity of HDL in preventing osteoporosis due to the lack of laboratory research on this topic. However, a low level of HDL is commonly linked to osteoporosis. A study carried out by Blair’s research groups in ApoA-1^−/−^ deficiency mice proved that the elimination of ApoA-1, the prominent component of HDL, leads to severe osteoporosis [17]. In these mice, the secretion of osteoblast-related factors such as RUNX2, Osterix, and COL1A1 is also reduced. Furthermore, in the same study, isolation of MSCs (mesenchymal stem cells) from ApoA-1^−/−^ deficiency mice showed that this cell prefers to differentiate into adipocytes rather than osteoblast cells. However, further analysis needs to be carried out to understand HDL’s beneficial activity on HOBs fully.

## 4. Materials and Methods

### 4.1. Culturing and Maintenance of HOBs 

Primary human osteoblast cells (HOBs) were purchased from Promocell (Heidelberg, Germany) at passage number 2. The cell tested positive for alkaline phosphates using the 5-bromo-4-chloro-3-indolyl phosphate (BCIP)/nitro blue tetrazolium (NBT) assay and positive for mineralization ability using Alizarin Red S. HOBs were grown and maintained in osteoblast growth medium containing 0.1 mL/mL (10%), fetal calf serum (FBS), and 1% Antibiotic-Antimycotic Solution, as recommended by the Promocell. Phosphate-buffered saline (PBS) (Sigma-Aldrich, St. Louis, MO, USA) was used as the washing solution and Accutase (Innovative Cell Technologies, San Diego, CA, USA) was used as the detachment solution during the sub-culturing. Cells in passages 6–7 were used for this study. Upon stimulation, the culturing medium was replaced with a treatment medium composed of DMEM media (ThermoFisher Scientific, Waltham, MA, USA) containing 15% FBS (Sigma-Aldrich, St. Louis, MO, USA), 10 mM β-glycerophosphate (Merck Millipore, Massachusetts, USA), 0.1 mM ascorbic acid (Sigma-Aldrich, St. Louis, MO, USA), and 1% Antibiotic-Antimycotic Solution (Sigma-Aldrich, St. Louis, MO, USA).

### 4.2. Oxidation of HDL

Human high-density lipoprotein (HDL) was purchased from Merck Millipore (Burlington, MA, USA). The HDL is certified to be from a healthy individual and the human plasma tested negative for HBsAg and HIV-I, HIV-II, HBc, and hepatitis C antibodies. Firstly, the HDL was dialyzed against phosphate-buffered saline (PBS) (Sigma-Aldrich, St. Louis, MO, USA) for 24 h in the dark at 4 °C with three buffer exchanges to remove any preservative agents. Then, about 1 mg protein/mL HDL was incubated with 50 µM copper sulphate (CuSO_4_) (Sigma-Aldrich, St. Louis, MO, USA) for 4 h at 37 °C. The mixture of HDL and CuSO_4_ was placed in a 15 mL centrifuge tube wrapped with aluminum foil and the incubation was performed in an incubator set at 37 °C. The oxidation was stopped by the addition of 2.5 mM EDTA (Sigma-Aldrich, St. Louis, MO, USA). The oxidized HDL was dialyzed again with PBS for 24 h in three buffer exchanges stored at 4 °C and used within 2 weeks. The value of TBARs in the oxHDL was measured using an OxiSelect™ TBARS Assay Kit (Cell Biolab Inc., San Diego, CA, USA) to evaluate the degree of oxidation. The average value of TBARs in this experiment was 119.9 ± 21 nmol/L/mg protein of MDA. 

### 4.3. Mineralization Detection via Alizarin Red Staining

An established Alizarin red S staining method was used to detect and quantify the mineralization of mineral nodules observed on the osteoblast cells [47]. About 35,000 cells/well HOBs were seeded overnight in a 24-well plate with treatment media. Then, the cells were induced with treatment media containing (i) media only as the negative control; (ii) 100 µM inorganic pyrophosphate (positive control); and (iii) 10, 25, 50, and 100 µg/Ml protein oxHDL in 400 µL of media (final total volume) for 14 days. The media was exchanged every 4 days. 

On day 14 of incubation, the media was removed, and the cells were washed three times with PBS. The cells were fixed by incubation with 100 µL of 4% formaldehyde (diluted in PBS) at 4 °C for 45 min. Next, the fixative reagent was removed, and the cells were washed with distilled water three times. Then, the cells were incubated with 1 Ml of 2% alizarin red (PH 4.2) for 20 min at room temperature with gentle shaking. The dye was removed, and the cells were gently washed twice with distilled water. Pictures of the cells were taken using an inverted microscope (Olympus, Tokyo, Japan).

In total, 800 µL of 10% acetic acid was added to each well and incubated at room temperature for 30 min with gentle shaking. The cells in each well were collected using a cell scraper, transferred into a 1.5 mL micro-centrifuge tube, and vortexed for 30 min. Then, the tubes were heated at 85 °C using a digital block heater for 10 min. The tubes were put on ice for 5 min. The tubes were then centrifuged for 15 min at 20,000× *g*. About 500 µL of the supernatant was transferred into a new micro-centrifuge tube. Then, about 200 µL of 10% ammonium hydroxide was added into each tube to neutralize the acidic solution. The standard curve of alizarin red was prepared by diluting the 4 Mm alizarin red in serial dilution. The Ph of each dilution was between 4.1 and 4.3. In total, 150 µL of the samples and standard curve dilution was transferred into a 96-well plate. The optical density (OD) was measured using a microplate reader (PerkinElmer 2030 Multilabel Reader VictorTM X5, Waltham, MA, USA) at a wavelength of 450 nm. The concentration of alizarin red obtained for each sample was calculated based on the standard curve.

### 4.4. Detection of Calcium Deposition Using the Calcium Colorimetric Assay

About 10,000 cells/well HOBs were seeded overnight in a 96-well plate in the treatment medium. Then, the cells were induced with a treatment medium containing (i) media only as the negative control; (ii) 100 µM inorganic pyrophosphate (positive control); and (iii) 10, 25, 50, and 100 µg/mL protein oxHDL in 100 µL of media (final total volume) for 14 days. The media was changed every 4 days. Then, on the 14th day of incubation, the cells were washed twice with PBS (phosphate-buffered saline). About 0.6 N HCL (100 µL) was added to each well to decalcify the cells. After 24 h, the supernatants were collected to quantify the calcium content using a Calcium Colorimetric Assay Kit (Sigma-Aldrich, St. Louis, MO, USA) while the protein content inside the cells was collected by solubilizing the cells with 0.1 N NaOH. The amount of calcium was normalized with the cells’ total protein content and quantified using a BCA protein assay kit (Thermo Fisher Scientific, Pierce, IL, USA).

### 4.5. Quantification of Bone Mineralization (ALPL, COL1A2, BGLAP, and RUNX2), Osteoblast-Related Transcription Factors (STAT-3 and PPAR-α), and Inflammatory (IL-6, TNF-α, RELA Proto-Oncogene, NF-kB Subunit (P65), and CREB) Markers Using Quantitative Reverse Transcription-Polymerase Chain Reaction (RT-qPCR) 

About 300,000 cells/well HOBs were seeded overnight in a 6-well micro-plate containing treatment medium. On the next day, the cells were incubated with (i) inorganic pyrophosphate (positive control); (ii) culture medium (negative control, denoted as the unstimulated group); (iii) HDL (100 μg/mL protein); (iv) oxHDL (100 μg/mL protein); (v) adiponectin (5, 10, and 15 μg/mL); and (vi) the combination of oxHDL (100 μg/mL) with adiponectin (5, 10, and 15 μg/mL). Total RNA was extracted using the AllPrep^®^ RNA/Protein kit (Qiagen, Hilden, Germany). The concentration and purity of the extracted protein and RNA were measured using a Nanodrop ND-100 Spectrophotometer. 

The total RNA extract samples were reverse transcribed to complementary DNA (cDNA) using an iScript™ gDNA Clear cDNA Synthesis Kit (Qiagen; Hilden, Germany) before the quantitative real-time PCR assay (rt-qPCR). Then, the cDNA was either stored at −20 °C or directly used as the template for the qPCR assay. SYBR-green was used as a detection method in the rt-qPCR assay. GAPDH and HPRT-1 were selected as the reference genes. The primer sequence for each measured gene is presented in Table 1. For every sample, 1 µL of cDNA was added to 9 µL of reaction mixture containing 1 µL of forward and reverse primer (400 nM final concentration), 5 µL of iTaq™ Universal SYBR^®^ Green Supermix (1 × final concentration), and 2 µL of DNase free water in a 10 µL volume and loaded into 96-well clear microtiter plates (Bio-Rad Laboratories, Hercules, CA, USA). Then, the reaction plates were sealed with optically clear adhesive films (Bio-Rad Laboratories, Hercules, CA, USA) before insertion into the CFX96TM Real-Time PCR Detection System (Bio-Rad Laboratories, Hercules, CA, USA) to perform PCR at 95 °C for 30 s (polymerase activation and DNA denaturation), 95 °C for 5 s (denaturation), 60 °C for 30 s (annealing/extension process), and repeated for another 49 cycles. Normal template controls (NTCs) and a reverse transcription control (RTC) were included in every reaction plate. A melt curve was obtained for each run to ensure the purity of the primer. The PCR efficiency was obtained by analyzing a series of cDNA (standard curve). Relative gene expression, ΔCT, was calculated using the formula: Ratio (references/target) = 2^Ct(references)−Ct(target)^ (Bio-Rad Laboratories, Hercules, CA, USA).

### 4.6. Statistical Analysis

The data were analyzed using Statistical Package for the Social Sciences (SPSS) version 21.0. The data were expressed as the mean ± SEM obtained from three experiments, where each assay was performed in triplicates (n = 3). A one-way ANOVA test was performed followed by post-hoc analysis (Bonferroni). The level of significance was set at *p* < 0.05. 

## 5. Conclusions

From this study, it can be concluded that inflammation is the link responsible for the occurrence of osteoblast demineralization induced by oxHDL. Even though HDL might not exhibit solid protective activity, it shows a better effect than oxHDL. Oxidation causes HDL to lose its anti-inflammatory properties, impairing its protective functions, where dysfunctional HDL starts to behave like a pro-inflammatory molecule. Interestingly, the detrimental effects of oxHDL could be suppressed by the presence of adiponectin. This shows that adiponectin may possess some beneficial activities. These findings could be fundamental for future development of drugs or treatments that could either protect HDL from being oxidized in the human body or drugs that could elevate the secretion of adiponectin.

## Figures and Tables

**Table 1:** Primer sequences of the target and reference genes.

Gene Name	Gene Symbol	Gene Bank Accession Number	Primer Sequence
Alkaline phosphatase	ALPL	NM_000478.5	F : CCAAGTACTGGCGAGACCAA
			R : TGTGGAGACACCCATCCCAT
Bone gamma-carboxyglutamate protein	BGLAP	NM_199173.5	F : TCACACTCCTCGCCCTATTG
			R : CTCTTCACTACCTCGCTGCC
Runt-related transcription factor 2	RUNX2	NM_0010246303	F : CCACCACTCACTACCACACC
			R : AAGGGTCCACTCTGGCTTTG
Collagen type I alpha 2 chain	COL1A2	NM_000089.3	F : GGATGAGGAGACTGGCAACC
			R : TGCCCTCAGCAACAAGTTCA
Interleukin 6	IL-6	NM_000600.4	F : TGAGGAGACTTGCCTGGTGA
			R : GCATTTGTGGTTGGGTCAGG
Tumor necrosis factor	TNF-α	NM_000594.3	F: GGTCCTCTTCAAGGGCCAAG
			R: TCCTCCTCACAGGGCAATGA
RELA proto-oncogene, NF-kB (p65) subunit	P65	NM_021975.3	F : AGGCTATCAGTCAGCGCATC
			R : TCCCCACGCTGCTCTTCTAT
cAMP responsive element binding protein 1	CREB1	NM_004379.4	F : CGAGAACCAGCAGAGTGGAG
			R : CGGTGGGAGCAGATGATGTT
Signal transducer and activator of transcription 3	STAT3	NM_139276.2	F : ACCATTGACCTGCCGATGTC
			R : GTGAGGGACTCAAACTGCCC
Peroxisome proliferator activated receptor alpha	PPAR-α	NM_001001928.2	F : AAGGCTGCAAGGGCTTCTTT
			R : ACATCCCGACAGAAAGGCAC
Glyceraldehyde 3-phosphate dehydrogenase	GAPDH	NM_002046.6	F : GGAGCGAGATCCCTCCAAAAT
			R : GGCTGTTGTCATACTTCTCATGG
Hyperparathyroidism 1	HRPT1	NM_000194_2	F : CCTGGCGTCGTGATTAGTGAT
			R : AGACGTTCAGTCCTGTCCATAA

## Data Availability

Not applicable.

## References

[1] Blair H.C., Larrouture Q.C., Li Y., Lin H., Beer-Stoltz D., Liu L., Tuan R.S., Robinson L.J., Schlesinger P.H., Nelson D.J. (2017). Osteoblast differentiation and bone matrix formation in vivo and in vitro. Tissue Eng. Part B Rev..

[2] Florencio-Silva R., Sasso G.R.D.S., Sasso-Cerri E., Simões M.J., Cerri P.S. (2015). Biology of bone tissue: Structure, function, and factors that influence bone cells. BioMed Res. Int..

[3] Finkelstein J.S., Elaine W.Y. (2022). Patient Education: Bone Density Testing (Beyond the Basics).

[4] Salari N., Ghasemi H., Mohammadi L., Rabieenia E., Shohaimi S., Mohammadi M. (2021). The global prevalence of osteoporosis in the world: A comprehensive systematic review and meta-analysis. J. Orthop. Surg. Res..

[5] Tarantino U., Cariati I., Greggi C., Iundusi R., Gasbarra E., Iolascon G., Kurth A., Akesson K.E., Bouxsein M., Tranquilli Leali P. (2022). Gaps and alternative surgical and non-surgical approaches in the bone fragility management: An updated review. Osteoporos. Int..

[6] Becker D.J., Kilgore M.L., Morrisey M.A. (2010). The societal burden of osteoporosis. Curr. Rheumatol. Rep..

[7] Byers R.J., Denton J., Hoyland J.A., Freemont A.J. (1997). Differential patterns of osteoblast dysfunction in trabecular bone in patients with established osteoporosis. J. Clin. Pathol..

[8] Demer L.L. (2002). Vascular calcification and osteoporosis: Inflammatory responses to oxidized lipids. Int. J. Epidemiol..

[9] Lacey D.C., Simmons P.J., Graves S.E., Hamilton J.A. (2009). Pro-inflammatory cytokines inhibit osteogenic differentiation from stem cells: Implications for bone repair during inflammation. Osteoarthr. Cartil..

[10] Feng X., McDonald J.M. (2011). Disorders of Bone Remodeling. Annu. Rev. Pathol..

[11] Parhami F., Morrow A.D., Balucan J., Leitinger N., Watson A.D., Tintut Y., Berliner J.A., Demer L.L. (1997). Lipid oxidation products have opposite effects on calcifying vascular cell and bone cell differentiation. Arterioscler. Thromb. Vasc. Biol..

[12] Tintut Y., Morony S., Demer L.L. (2004). Hyperlipidemia promotes osteoclastic potential of bone marrow cells Ex Vivo. Arterioscler. Thromb. Vasc. Biol..

[13] Mazière C., Savitsky V., Galmiche A., Gomila C., Massy Z., Mazière J.C. (2010). Oxidized low density lipoprotein inhibits phosphate signaling and phosphate-induced mineralization in Osteoblasts. Involvement of oxidative stress. Biochim. Biophys. Acta Mol. Basis Dis..

[14] Ito F., Ito T. (2020). High-density lipoprotein (HDL) triglyceride and oxidized HDL: New lipid biomarkers of lipoprotein-related atherosclerotic cardiovascular disease. Antioxidants.

[15] Srivastava R.A.K. (2017). Dysfunctional HDL in diabetes mellitus and its role in the pathogenesis of cardiovascular disease. Mol. Cell. Biochem..

[16] Rosenson R.S., Brewer H.B., Ansell B.J., Barter P., Chapman M.J., Heinecke J.W., Kontush A., Tall A.R., Webb N.R. (2015). Dysfunctional HDL and atherosclerotic cardiovascular disease. Nat. Publ. Group.

[17] Blair H.C., Kalyvioti E., Papachristou N.I., Tourkova I.L., Syggelos S.A., Deligianni D., Orkoula M.G., Kontoyannis C.G., Karavia E.A., Kypreos K.E. (2016). Apolipoprotein A-1 regulates Osteoblast and lipoblast precursor cells in mice. Lab. Investig..

[18] Rahim S., Abdullah H.M.A., Ali Y., Khan U.I., Ullah W. (2016). Serum Apo A-1 and its role as a biomarker of coronary artery disease. Cureus.

[19] Martineau C., Martin-Falstrault L., Brissette L., Moreau R. (2014). The atherogenic Scarb1 null mouse model shows a high bone mass phenotype. Am. J. Physiol. Endocrinol. Metab..

[20] Isales C.M., Zaidi M., Blair H.C. (2010). ACTH is a novel regulator of bone mass. Ann. N. Y. Acad. Sci..

[21] Papachristou N.I., Blair H.C., Kypreos K.E., Papachristou D.J. (2017). High-Density Lipoprotein (HDL) metabolism and bone mass. J. Endocrinol..

[22] Kirk B., Feehan J., Lombardi G., Duque G. (2020). Muscle, bone, and fat crosstalk: The biological role of myokines, osteokines, and adipokines. Curr. Osteoporos. Rep..

[23] Wang Z.V., Scherer P.E. (2016). Adiponectin, the past two decades. J. Mol. Cell Biol..

[24] Lubkowska A., Dobek A., Mieszkowski J., Garczynski W., Chlubek D. (2014). Adiponectin as a biomarker of osteoporosis in postmenopausal women: Controversies. Dis. Markers.

[25] Pierce J.L., Begun D.L., Westendorf J.J., McGee-Lawrence M.E. (2019). Defining osteoblast and adipocyte lineages in the bone marrow. Bone.

[26] Hyun W.L., Sang Y.K., Kim A.Y., Eun J.L., Choi J.Y., Jae B.K. (2009). Adiponectin stimulates Osteoblast differentiation through induction of COX2 in mesenchymal progenitor cells. Stem Cells.

[27] Luo X.H., Guo L.J., Yuan L.Q., Xie H., Zhou H.D., Wu X.P., Liao E.Y. (2005). Adiponectin stimulates human Osteoblasts proliferation and differentiation via the MAPK signaling pathway. Exp. Cell Res..

[28] Komori T. (2009). Regulation of osteoblast differentiation by Runx2. Osteoimmunology.

[29] Li J. (2013). JAK-STAT and bone metabolism. Jak-Stat.

[30] Proshchalykin M.Y., Dathe H.H. (2012). Peroxisome proliferator-activated receptor activators modulate the osteoblastic maturation of MC3T3-E1 pre-osteoblasts. Zootaxa.

[31] Baum R., Gravallese E.M. (2014). Impact of inflammation on the osteoblast in rheumatic diseases. Curr. Osteoporos. Rep..

[32] Kao R., Lu W., Louie A., Nissenson R. (2012). Cyclic AMP signaling in bone marrow stromal cells has reciprocal effects on the ability of mesenchymal stem cells to differentiate into mature osteoblasts versus mature adipocytes. Endocrine.

[33] Langdahl B., Ferrari S., Dempster D.W. (2016). Bone modeling and remodeling: Potential as therapeutic targets for the treatment of osteoporosis. Ther. Adv. Musculoskelet. Dis..

[34] Orimo H. (2010). The mechanism of mineralization and the role of alkaline phosphatase in health and disease. J. Nippon Med. Sch..

[35] Chang J., Liu F., Lee M., Wu B., Ting K., Zara J.N., Soo C., Al Hezaimi K., Zou W., Chen X. (2013). NF-κB inhibits osteogenic differentiation of mesenchymal stem cells by promoting β-catenin degradation. Proc. Natl. Acad. Sci. USA.

[36] Domazetovic V., Marcucci G., Iantomasi T., Brandi M.L., Vincenzini M.T., Vincenzini M.T. (2017). Oxidative stress in bone remodeling: Role of antioxidants. Clin. Cases Miner. Bone Metab..

[37] Kaneshiro S., Ebina K., Shi K., Higuchi C., Hirao M., Okamoto M., Koizumi K., Morimoto T., Yoshikawa H., Hashimoto J. (2014). IL-6 negatively regulates osteoblast differentiation through the SHP2/MEK2 and SHP2/Akt2 pathways in vitro. J. Bone Miner. Metab..

[38] Ambrogini E., Que X., Wang S., Yamaguchi F., Weinstein R.S., Tsimikas S., Manolagas S.C., Witztum J.L., Jilka R.L. (2018). Oxidation-specific epitopes restrain bone formation. Nat. Commun..

[39] Baker R.G., Hayden M.S., Ghosh S. (2011). NF-κB, inflammation, and metabolic disease. Cell Metab..

[40] Chang J., Wang Z., Tang E., Fan Z., Mccauley L., Franceschi R., Guan K., Krebsbach P.H., Wang C.Y. (2009). Inhibition of osteoblast functions by IKK/NF-κB in osteoporosis. Nature.

[41] Boyce B.F., Yao Z., Xing L. (2010). Functions of NF-κB in Bone. Ann. N. Y. Acad. Sci..

[42] Li Y., Li A., Strait K., Zhang H., Nanes M.S., Weitzmann M.N. (2007). Endogenous TNFα lowers maximum peak bone mass and inhibits osteoblastic Smad activation through NF-κB. J. Bone Miner. Res..

[43] Duttenhoefer F., Biswas S.K., Igwe J.C., Sauerbier S., Bierhaus D.D.S.A. (2014). Sp1-dependent regulation of PPAR α in bone metabolism. Int. J. Oral Maxillofac. Implant..

[44] Zhang Z.R., Leung W.N., Li G., Kong S.K., Lu X., Wong Y.M., Chan C.W. (2017). Osthole enhances osteogenesis in Osteoblasts by elevating transcription factor Osterix via cAMP/CREB signaling in vitro and in vivo. Nutrients.

[45] Nishihara S., Ikeda M., Ozawa H., Akiyama M., Yamaguchi S., Nakahama K.I. (2018). Role of cAMP in phenotypic changes of Osteoblasts. Biochem. Biophys. Res. Commun..

[46] Itoh S., Udagawa N., Takahashi N., Yoshitake F., Narita H., Ebisu S., Ishihara K. (2006). A critical role for interleukin-6 family-mediated Stat3 activation in Osteoblast differentiation and bone formation. Bone.

[47] Gregory C.A., Gunn W.G., Peister A., Prockop D.J. (2004). An Alizarin red-based assay of mineralization by adherent cells in culture: Comparison with cetylpyridinium chloride extraction. Anal. Biochem..

